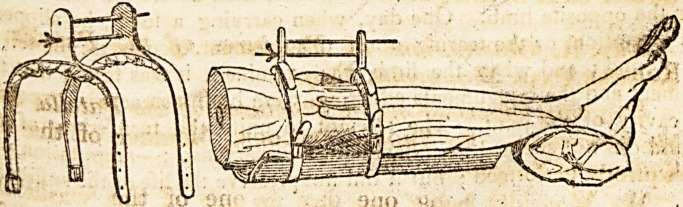# Transactions of the Associated Apothecaries

**Published:** 1824-06-01

**Authors:** 


*Transactions of the Associated Apothecaries and Surgeon-Apothecaries of England and Wales.* Vol. 1, pp. 424, with plates, 1823.

In preceding Numbers of this Journal, we brought down the analyses of the various articles as far as p. 272, of the volume, and we now mean to embrace the whole of the papers from that to the end of the work, in our present number.

Art. I. *Observations on a Series of Cases of Inflammatory Diarrhœa, occurring in the Winter of* 1820-21, *and apparently caused by Congestion and Obstruction of the Liver, joined to an Irritated and Inflamed State of the Mucous Membrane of the Intestinal Canal.* By C. T. Haden.

Mr. Haden observes that, in this country, bowel-complaints appear to occur under opposite states of the atmosphere, putting on a corresponding opposition of character. It is rare, he thinks, that they exhibit inflammatory symptoms in autumn; while inflammation forms, as it were, the essential character of those of winter. In the autumn, the violence of the vomiting and purging reduces the patient to a state of great weakness—in the winter, the inflammatory symptoms are too evident to be mistaken. Still he thinks the cause of both complaints is the same, though the symptoms and treatment vary. He thinks inflammation, or something akin to this, is at the bottom of all. As we can glean little from this paper, which is at all beyond the common run of observation, we must be excused from dwelling farther on it in this place.

Art. II. *Observations on Fractures of the Patella.* By Robert Palk Mogridge, Esq.

*Practical Observations on Fractures of the Patella and the Olecranon.* By Thomas Alcock, Member of the Royal College of Surgeons.

Mr. Mogridge being one day in one of the wards of St. Thomas's Hospital, a man was brought in with fracture of the patella. The dressers were proceeding to bandage it, on Sir

Everard Home's plan. A discussion took place, but Sir Everard's method was carried into effect.

"A wide tape roller was bound very tight round the limb above the knee, and the superior fractured portion of the patella; another below the knee, and inferior portion; under these were placed other slips of tape, coming about two inches beyond each bandage, one on each side of the patella, one on each side of the knee, and one directly over the patella. The upper ends of all these were turned down and pinned to the superior bandage, then the inferior ends were pulled upwards as tightly as possible, so as to bring the rollers as nearly together as practicable, and with them the separated portions of bone. At first view I perceived that this could not answer, for the bandages themselves were tighter than the patient could well bear, before the bracing of the slips; therefore, we may at once say, that the more insufferable, the better the effect of this plan. By this mode of reasoning I therefore concluded, that the plan was bad, as it afterwards turned out, for the patient could not bear the torture, so that the bandages were loosened continually, and thus the effect destroyed: he always complained of extreme coldness in the extremity, which is not to be wondered at when the circulation must be almost stopped, and so much pressure on the nerves." 287.

Mr. M. now set about devising some better apparatus. He had heard Sir Astley Cooper remark in his lectures, that he did not doubt the possibility of osseous union of the fractured portions of patella, provided they could be held in contact for a certain time. In any instrument for this purpose, Mr. M. observes, there must be great pressure on the superior portion of the tibia and inferior portion of the femur, before the screw should act. The pressure on the ham too must be entirely done away with; for by preventing free circulation, the union is retarded.

"Both these difficulties I surmounted, by having a very thick splint made, so that the pressure should be on the calf of the leg and the thigh: by this arrangement the effect of the pressure becomes immediately reversed; it now does good, for, the more pressure you apply, the straighter the leg is brought, and without increase of pain. Having so far succeeded in obtaining the principles, I tried to apply them, and produced the instrument, of which I shall now give a description, accompanied necessarily by a sketch, together with the mode of its application.

"The part of this instrument which presses on the knee is of highly tempered steel, with a well and hard stuffed cushion underneath, the leather strap covering it above: into this are firmly rivetted the upright pieces, through which the screw and guide pass. No instrument can be more simple. I have given no plan for the splint, as it is but a thick common one. The method of applying it is by unscrewing the thumb-screw to its extent, then getting the portions of fractured bone between the instrument; apply the splint as represented in the annexed figure, buckle the strap around the limb and splint as tight as possible, then with the thumb-screw bring the instrument together. I have applied it on my own leg until I have held the patella as tight as if it had been squeezed by a blacksmith's vice. The great advantage of it is, that the patient wants no attendance after the first application, as he can tighten it himself, and I should think he can walk with it. The guide is absolutely necessary, as without it the screw could not work: it is merely a steel wire rivetted at one end, and passing through a hole at the other: nothing but lengthening the upright, and forming a hinge as a pair of compasses, could be substituted, and this would be inconvenient from the length. Mr. Smith, an ingenious instrument-maker, living in St. Saviour's Churchyard, Borough, made this instrument under my direction; he has one at present in his possession." 289.

As far as one can judge from appearances, and before the actual application of the apparatus to a case of fracture, the plan seems feasible. Beyond this we dare not go.

Mr. Alcock remarks that the serious evils attending fractures of the patella, when ossific union is not produced, and the discrepancy of opinion among eminent surgeons respecting the mode of treatment, render further investigation of the subject necessary. Mr. A's attention was drawn to this accident by a case that made a deep impression on his mind at the time.

"A man, in an humble and laborious station in life, had suffered the common fracture of the patella, from which he had so far recovered as to resume his employment; but the union of the fractured portions of the patella was not by osseous matter, but by a considerable extent of ligament; so that the broken ends were, after the cure (if cure it can be called), far from being in contact. To those who have observed the effects of a ligamentous union after fracture of the patella, it need not be stated that this limb remained much weaker than before the accident, or than the opposite limb. One day, when carrying a load, he slipped; and laceration, or the tearing up of this ligament, occurred: he fell to the ground; and when the limb was examined, it was found that the ligament and the integuments adhering, were both torn across, exposing the cavity of the joint. The attempt to unite the torn edges of the wound did not succeed;—inflammation supervened;—amputation was subsequently performed; but it did not preserve the life of the sufferer." 291.

Our author witnessed another case nearly as distressing as the above. A female suffered fracture of the patella, which united by ligament. Some months afterwards the same accident happened to the other patella, with similar union. After a long confinement the poor creature was discharged, but unable to walk without crutches. We lately heard of a case with a very different result. A man had fracture of the patella, which united by ligament, and he went lame. Some time afterwards he fractured the other patella, after recovering from which he walked as well as ever he did in his life—greatly rejoiced at the second accident, which put both extremities on an even footing.

Mr. Alcock avers, that in his own practice, and that of others, he has seen many cases, where "perfect ossific union of the patella took place." In a note, however, while alluding to the scepticism of some surgeons on this point, Mr. A. remarks that—"he is not anxious for the term (ossific union) and candidly states that he is indifferent by what name the union be designated, provided it is so perfect, that the injured part be equally strong and useful as before the fracture."

If we examine a recent fracture of the patella we find, of course, that the lower portion remains *in situ*, being attached to *ligament*, while the upper portion is necessarily drawn up by the action of the large *muscles* inserted into it. To counteract this retraction, our author avers, that little more is necessary than bringing the leg in a right line with the thigh, and this last raised towards the front of the pelvis—or otherwise the pelvis bent forwards on the thigh. "In practice, says Mr. A. it will be found that this position nearly accomplishes the indication of keeping the broken ends of the bone together; or at least renders the slightest force sufficient, when properly directed, to retain them steadily in contact."

"The muscles having been relaxed by the position above alluded to, let the surgeon compress the broken portions of the bone gently between his fingers and thumbs, using the fingers to one portion and the thumbs to the other, increasing the pressure until the upper portion be in perfect contact with, and apposition to the lower. Let him observe the extent of force which is necessary to effect this accurate apposition; and he will find, that a force equivalent to a few ounces in weight will suffice; if the relaxed position of the muscles have been well observed. Let those who may be of opinion that the aid of the mechanical powers is required to effect this simple purpose, examine well this part of the treatment, and if a doubt remain, rather remove the support of the fingers from the upper portion, and again observe how slight a force will suffice to bring it back to its natural position; for, in truth, no pain nor inconvenience will be experienced from any part of the treatment, unless the force used exceed the necessity of the case; and the patient, so far from complaining, will be more apt to express his satisfaction at the comfortable degree of support which either the hand, or the apparatus, subsequently supplying its place, affords him." 295.

The following directions for the management of this fracture we shall give in Mr. Alcock's own words.

"The apparatus may be very simple: the writer has generally used strips of plaister of about an inch in breadth and a foot long, crossing obliquely from the integuments immediately above the patella to the upper and back part of the leg, the patella being within the angle formed by the crossing. This, he has believed, rendered the bandage and compress less liable to slip, but he does not consider the plaister essential. A moderate-sized compress has been then placed immediately above the patella, the ends bending downwards on each side, so that the bandage has rested upon it, and has produced an equable and steady, though moderate compression, in a direction opposite to that of the extensor muscles; thereby counteracting any contraction which, under the previously detailed circumstances, they may be likely to exert. A narrow double-headed flannel bandage has been generally preferred, on account of its greater elasticity than linen or calico. A splint may or may not be placed in the ham. If the steadiness of the patient can be depended upon, the splint may be dispensed with;—if his steadiness be doubtful, the splint had better be used. The bandage may be applied in any convenient manner, forming a sort of fulcrum by the use of pins whenever it becomes necessary to change the direction of the bandage, so as to make it bear particularly upon any required point. It is not likely that any one expert in the use of the roller, and having a clear idea of the object to be attained by its application, will fail in giving the necessary support where it is required. The bandage should not be so tight as to cause the leg to swell, otherwise the lower part must be also supported. There is an advantage in leaving the patella uncovered, as it enables the surgeon not merely to suppose that the ends of the bone are steadily supported in contact, but to assure himself of the fact, day by day, without disturbing the apparatus; unless any slipping of the bandage, or slight retraction of the upper portion of the bone, should render it necessary." 297.

After the first few days it may be ascertained that slight flexion of the knee may be allowed, to relieve irksome feelings, without deranging the apposition of the bones. After this the patient may be allowed to move about upon crutches, supporting the injured limb in a broad sling passed over the shoulders. At the end of a month the foot may be put cautiously to the ground, but the knee is not to be bent.

From fracture of the patella the transition is natural to that of the olecranon. This last, however, is always the result of external injury. The mode of union is generally by ligament, elongated in proportion to the retraction of the broken portion of bone. "In this instance the power of extending the fore-arm is greatly diminished; whilst the natural support afforded by the extremity of the bone to prevent the too great extension of the forearm being lost, the forearm may, by external force, be carried backwards beyond the direct line of the humerus."

"Another mode of union takes place when the broken portion has not been kept sufficiently near to the part from which it has been separated; yet not so distant as to prevent ossific union. Consequently the extremity of the bone projects further than natural, the forearm cannot be fully extended, and considerable inconvenience and lameness result. When this mode of union occurs, there is frequently great irregularity and enlargement of the bone at the place where it has united."

The third and (according to our author) most desirable termination is when, by great care, a perfect osseous union is effected. This Mr. A. thinks may generally, if not always, be obtained.

"It is obvious that the simple principles of practice in fracture of the olecranon are, to diminish the swelling which the violence necessary to produce fracture generally occasions;—to guard against inflammation;—to replace the fractured portion, and keep it steadily in its natural position; to relax the muscle (the triceps) inserted into it;—to prevent rigidity of the joint by appropriate exercise, as soon as the union becomes sufficiently firm to admit of it with safety; &c.—but an ordinary example may supply the place of further detail.

"Sept. 20, 1820. A young man, aged twenty, was thrown out of a gig, and fell upon his right elbow. His face and right hip were also injured. The swelling of the elbow was so great, as to prevent the examination by the touch being satisfactory; although the inference was clear, from the manner in which the accident had occurred, and the extent of injury around the elbow, that the olecranon must have suffered fracture. Treatment—bleeding, both general and topical; purging; low diet, and cold applications to the injured parts; to rest the arm.

"Sept. 24. The swelling of the elbow was so much reduced as to admit the existence of fracture to be distinctly ascertained. The olecranon was broken off and drawn upwards. The fracture was reduced; and the detached part kept down by compress, adhesive strips, and bandage; the arm put in the extended position, and a hollowed splint placed in front of the elbow joint, to prevent accidental flexure.

"Oct. 4. No pain. 'There is no crepitus now perceptible. The broken portion is perfectly in place, and resists the slight force which can be prudently used in examination.'

"6th. Arm firmer;—bandage adjusted.—It is needless to state the daily progress. The olecranon united so perfectly in its natural place, that it required careful examination to distinguish it from that which had not been injured. For some time after leaving off the splint the motion of the joint was checked, no doubt from having been kept stationary in the extended position; but by daily using, at first, passive flexion; and afterwards, swinging the forearm with a small weight in the hand, the use of the joint was perfectly restored. Friction was combined with this exercise. He was perfectly well by the end of October." 304.

Art. III. *Some Observations on the Utility of Opium in Certain Inflammatory Disorders.* By John Armstrong, M.D. Lecturer on the Principles and Practice of Physic.

A great deal of theoretical prejudice has obtained, and still obtains, respecting the physiological effects of opium on the human frame. Its action has been too generally and too strictly looked upon as stimulant, without taking into just consideration its other properties. Practitioners in the hotter regions of the earth, and especially in India, have long been in the habit of giving opium freely in acute diseases, and even in topical inflammation, after or in company with, venesection; and generally in combination with calomel. The utility of this practice has been long established, in those climates, and it is now making its way in this country, with some little variety in the modus.

Some years ago the illustrious author of the paper before us had his attention drawn to the subject in question, by observing a chasm or defect in the common modes of treating acute abdominal inflammation by the simple depletion of bleeding and purging. He had observed long before that period, that when opium was given *in full doses* immediately after copious depletion, the cases terminated successfully.

"Under this impression, I determined to administer opium in future more boldly, in those cases which appeared most promising for its favourable effects. Within the last four years, I have prescribed large doses of opium, conjointly with blood-letting, in at least a hundred cases of acute and sub-acute abdominal inflammation, proceeding from common causes; and as its efficacy has considerably exceeded that of any other remedy tried under similar circumstances, I shall endeavour to point out in this paper, first, those circumstances, and secondly, the most efficacious doses." 310.

The following symptomatic, or pathognomonic sketch we shall give in our author's own words.

"Acute inflammation of the peritoneal coat of the bowels is generally marked by a distinct pain in some part of their course, increased under pressure, and attended by a quick, small, hard pulse, a hurried respiration, a hot skin, a whitish moist tongue, flatulence, constipation; and nausea, retching, or vomiting occur, if not always at its commencement, at least during its progress. When this form of inflammation is seated in the peritoneal coat of the stomach, the symptoms are similar, except that the pain is limited, not to some part of the intestines, but to the epigastric region, while the pulse is smaller, and the vomiting usually urgent from the beginning. In acute peritonitis the pain is diffused over the abdomen, the pulse is fuller, the heat higher, and the stomach is seldom disturbed by nausea, retching, or vomiting, till towards the close of the disease. The uterus is rarely inflamed acutely, except after delivery, and the inflammation is denoted, then, by a hard circumscribed tumour in the hypogastric region, painful on pressure, and attended by much fever; but in such instances it frequently happens, that the peritoneum itself is inflamed, or the peritoneal coat of the intestines, and then the symptoms have a mixed character. In acute nephritis there is, on one or both sides of the loins, a distinct pain, increased by pressure applied forcibly there, and on the directly opposite side of the belly. More or less pain or retraction of one of the testes, scanty urine, and fever, are the concomitants. Many symptoms have been enumerated as pathognomonic of acute hepatitis, but the only one upon which I would rely is pain on pressure in the region of the liver, accompanied by fever; though the colour of the stools, urine, or skin, occasional chills, depression of spirits, and other signs, will unquestionably assist in the diagnosis." 311.

In acute inflammation of the peritoneal coat of the stomach or bowels, Dr. A. makes it a rule to see the patient bled, in the first stage, to complete relaxation—approaching syncope, whatever may be the quantity necessary to produce this effect. As soon as ever the patient recovers from the faintness, three grains, at least, of good opium, in the form of a *soft* pill, are given, and quietude is strictly enjoined, so that, if possible, sleep may be obtained. In some irritable habits less of the solid, and some fluid opium are prescribed, in order that the anodyne and sedative effects may be more quickly produced.

"The effects of opium thus administered, are to prevent a subsequent increase in the force or frequency of the heart's action, and a return of the abdominal pain, while it induces a tendency to quiet sleep, and a copious perspiration over the whole surface. In many instances, this simple procedure will remove the inflammation at once, nothing being afterwards necessary, when the patient awakes, but spare diet, absolute rest and quietness, with an occasional mild laxative. But on all occasions, if possible, I visit the patient about three or four hours after the administration of the opium, and if there be pain on pressure in any part of the abdomen, with a hot skin, and quick jerky pulse, I order the patient, in my presence, to be promptly bled again in the same decisive manner as before." 312.

Our author properly observes that some physicians commit a great mistake by dictating on paper the quantity of blood to be drawn. We hope, for the honour of medical science, that this is now very rarely done—at least we have not seen any physician so absurd for some years past. Dr. A. is perfectly right in averring that "it is solely upon the *effect* produced that the benefit of blood-letting depends"—but we cannot entirely acquiesce in the remaining portion of the sentence—"and therefore the effect should always be witnessed by the physician." If the general practitioner who bleeds the patient were a mere phlebotomist, or a chemist's apprentice, we would say Dr. Armtrong was fully justified in the above precaution; but knowing, as we do, the education and the practical information diffused, in our days, among the general practitioners, we have no hesitation in avowing, that there is rarely much danger in trusting to the judgment of surgeon-apothecaries upon such occasions, since they are equally aware with the physician of the great importance attached to effectual depletion in the early stages of abdominal, and indeed of all acute inflammations.

After this second abstraction of blood carried again to complete relaxation, our excellent author generally prescribes about two grains of opium with three or four grains of calomel, exhibited in the form of a pill, as the faintness disappears. The patient is again left in perfect quietness, and refreshing sleep with free perspiration most frequently succeeds. A third venesection is rarely requisite; but if, after the expiration of five or six hours from the second, pain and fever still exist, the operation should again be performed as before; and one grain of opium with two or three grains of calomel given almost immediately afterwards—while half a grain of opium and two of calomel may be repeated every four hours till sleep and general perspiration be induced.

"It is repeatedly observed in my works, and the observation was made long before their appearance, that the specific effects of mercury are easily procured when large quantities of blood are abstracted under its administration. For this reason, the calomel should be given with proportionate care, whenever copious and repeated blood-letting becomes necessary." 313.

The above plan, with the exception of giving a large dose of opium alone, after the first bleeding, is nearly that which we have ourselves pursued for twenty years past, and which has been pursued by most tropical practitioners. Dr. Armstrong's modification of it we think a decided improvement, from some trials which we have lately made, and therefore we recommend it to the consideration of our professional brethren.

Dr. A. observes, that when the cure has been left entirely to his own management, he has never found it necessary to bleed more than thrice, in the most severe examples of acute inflammation; though now and then the additional aid of leeches to the abdomen has been deemed expedient. We may just remark, *en passant*, that we do not quite agree with our able author on the point of local blood-letting. We attach more importance to this measure than Dr. A. seems to do. We have often found, that when the vascular action was brought down by the venesection carried to syncope, the slow but steady drain from twenty or thirty leech bites *kept down* the excitement, and thus prevented the necessity for further venesection. On the subject of purgatives, every practitioner of observation must agree with Dr. Armstrong. The best way to open the bowels is by bleeding. Constipation is not the cause but the effect of the inflammation, and to remove the cause is the first object. A discharge from the bowels very frequently follows the bleeding; and if not, the bowels are easily opened when the inflammatory tension is taken off the peritoneal covering. Large glysters of warm water, however, are always proper for the purpose of removing accumulated fæces from the colon, and inviting discharges from the upper intestines.

"Large and repeated doses of opium tend to lock up the secretion of the liver, and therefore, in acute hepatitis, they should rarely be repeated beyond the second time, being always premised by venesection, and always conjoined with calomel. Moreover, saline purgatives should be freely employed from the beginning, and if any traces of inflammation should be left, in despite of active evacuations, the mouth ought to be affected by mercurials. A similar plan may be pursued in common peritonitis and nephritis. In the first and subsequent editions of the Illustrations of Typhus and other febrile Diseases, a striking case of the latter is detailed, in which full doses of opium, united with calomel, succeeded even when copious venesection had failed; and I may here add, that I have since witnessed some cases of inflammation of the bowels, where full doses of opium finally effected the cure, after bleeding and purging had completely disappointed my expectations. So great indeed is my confidence in full doses of opium in peritoneal enteritis, that if compelled to say, supposing myself the subject of the disorder, whether I would exclusively rely upon them solely, or upon blood-letting solely, I should certainly fix upon the former; at the same time I should like to have the simultaneous influence of both remedies, being convinced, that they are far more serviceable combinedly, than separately employed." 315.

We confess that did we labour under enteritis, and had only the choice of opium or the lancet, we should not hesitate to prefer the *latter*—but every man to his taste.

Dr. A. sometimes gives larger doses of the opium than above stated, but never beyond five grains. He remarks that, in some instances, where all signs of abdominal inflammation have subsided, the pulse continues considerably quicker than natural, and a simple fever ensues. So long as this fever lasts the patient must be kept in bed, the diet must be spare, the bowels kept open, an opiate administered at bed-time. Our author has discarded from his practice "the employment of such medicines as digitalis, prussic acid, and tartarized antimony, in the beginning of acute inflammations." The advantage of tartarized antimony as an auxiliary to the lancet and opium will, we believe, be acknowledged by most practitioners who have attentively watched the operation of the remedy. At the same time it is proper to remark that antimony is far less applicable to inflammations of the abdominal than of the thoracic viscera, on account of the gastric irritability so commonly attendant on the *former* class of complaints.

"As soon as I had satisfactorily ascertained the combined efficacy of blood-letting and opium in acute abdominal inflammations, I mentioned the results of my experience privately and publicly in the metropolis. Several practitioners have tried this treatment, and, so far as I have yet heard, found it similarly beneficial. It has now been employed extensively by myself and others in those acute forms of abdominal inflammation which so often follow delivery, and in which it has been more uniformly efficacious than any other. The great peculiarity of acute abdominal inflammation in the puerperal state is, that it runs a more rapid course than ordinary, and therefore requires to be more promptly subdued. Though in the country my success was considerable in what is vaguely called puerperal fever, yet under the same treatment in London, namely, bleeding and purging, I am fully persuaded that a great many patients would have been lost. Women are much more irritable in London than in the country, probably on account of their more sedentary and artificial habits; and by consequence they are much more liable to that reaction of the heart and general irritation, which are so apt to follow copious bleeding in them, and which appear to renew the inflammation when allowed to advance, but which may almost invariably be controlled, by full doses of opium given at the precise juncture before mentioned." 318.

We fear, from recent facts, that puerperal fever has resisted this and every other mode of treatment with as much obstinacy as at any former period of its history.

Dr. A. observes, that as opium has a specific effect on the vessels of the head, great care is necessary in its exhibition, when the brain is affected. A moist tongue, Dr. A. conceives, is essential to the good effects of opium—"and therefore, in specific fevers, such as typhus, where the tongue is dried and glazed, it always does harm instead of good, even where abdominal inflammation is present." The only cases where our author has known opium beneficial while the tongue was dry, were those which had been preceded by copious hæmorrhage, and certainly in many of these it has (he avers) apparently saved the patient by allaying the existing irritation, and preventing the occurrence of that violent reaction of the heart, by which the hæmorrhage is so liable to be renewed.

"In several cases of acute inflammation of the pericardium, of the pleura, and of the substance of the lungs, I have tried the large doses of opium after copious venesection, with similar benefit as in the acute abdominal inflammation before mentioned; but it is a practice which I would not be understood to recommend in inflammation of the mucous membrane of the bronchia, an affection which requires, in many instances, the greatest circumspection as to blood-letting, and in which those measures which act simultaneously on the bowels and on the skin are singularly useful. Where the heat on the surface is universally high in bronchitis, and the pulse at the same time expanded and resisting, I have found moderate venesection very serviceable; but when the heat is subdued, and the pulse small and compressible, I have generally avoided it altogether, and trusted to the forementioned means, with an antiphlogistic diet, and a regulated temperature. One of the leading advantages of what might be called anatomical physiology is the ascertainment of the different structures and functions of adjacent parts; and another of the leading advantages of what might be called anatomical pathology is the different results which are displayed, by an accurate examination of those parts after death. But minute observation of the rise, progress, and decline of the symptoms, together with an exact register of the effects of remedies at these different periods, are still necessary to enable us to turn our anatomical physiology, and our anatomical pathology to great practical account. It appears to me, that the French, generally speaking, excel the English in anatomical physiology, and in anatomical pathology; but it also appears to me equally certain, that they have not observed either the symptoms, or the effects of remedies so accurately as we have done, and the English therefore really excel them in the precise application of remedies. But this remark is only referrible to those physicians in this country, who, observing and thinking for themselves, merely deem symptoms the indications of disease, and strive to connect them, as closely as possible, with the condition of different parts of the body upon which they depend; for it must be admitted, that those practitioners who still pursue the nosological method of affixing to certain symptoms an abstract name without a knowledge of the condition with which they are connected,—it must, I repeat, be admitted, that the practice of such men is mere empiricism, similar to that which the public passively adopt and dangerously apply from tradition." 320.

The observations of Dr. Armstrong on the French pathology and practice in the above passage, we are happy to find in unison with what we have all along insisted on in this journal.

Our author has found full doses of opium after copious bloodletting, cut short inflammation of the mucous membrane of the intestines.

"Sub-acute inflammation of the mucous membrane, especially of that portion which invests the small intestines, is exceedingly common as an original affection in this country, both among children and adults. It is generally denoted by an obscure pain in some part of the abdomen increased under pressure, and accompanied by a quick soft pulse, a hottish skin, a slightly furred tongue remarkably red at the top, and a short way thence round the edges; while the stools, from an increased mixture of mucus, most frequently have an oleaginous sort of consistence, and are somewhat darker and more offensive than natural. In the London Fever Hospital I have had a great many opportunities of pointing out this particular form of inflammation to my pupils, and also of shewing the great efficacy of small or moderate doses of calomel conjoined with a few grains of rhubarb, and assisted by a little cold-drawn castor oil. The French pathologists have overlooked the general connexion which a disordered state of the liver has with sub-acute inflammation of the mucous membrane of the intestines. Wherever this connexion exists, small or moderate doses of calomel, united with mild laxatives, will be found highly useful, seemingly by gently dislodging the morbid accumulations in the bowels, and particularly by increasing a flow of bile, from which, probably, the blood finds a readier access through the liver, and thus influences the circulation of the splenic, the superior and inferior mesenteric veins, and their ramifications. In all cases, however, of this complicated nature, I have applied leeches to the abdomen, and repeated them as long as there was any pain on pressure; and experience has taught me that they may be employed preferably to general blood-letting in most sub-acute inflammations of the mucous membrane of the bowels. In such examples, the blandest and sparest diet is necessary, for any deviation in that respect is apt to maintain the inflammation, in defiance of the best remedies." 321.

The paper, our readers must perceive, will not detract from the high reputation of Dr. Armstrong, as an accurate observer, enlightened practitioner, and ingenious reasoner.

ART. IV. *Case of blighted Ovum.* By J. HAYES, Member of the Royal College of Surgeons, and of the Society of Apothecaries.

In her fourth pregnancy Mrs. H—— engaged the professional services of our author for her next accouchement. This was about the middle of utero–gestation. In a fortnight afterwards he was sent for, and found the lady complaining of pain at the bottom of the belly, extending down the thighs, having some coloured discharge from the vagina, and being much agitated in mind. She had been frightened. Quietude, venesection—an aperient—afterwards an anodyne. Next day the discharge had nearly ceased—the pain much alleviated—and she was altogether better. Quietude in bed prescribed. Third day. Had felt an unusual coldness and sense of weight in the belly, and also a severe shivering fit, which lasted half an hour, but was not succeeded by correspondent reaction. She quite recovered in a few days. Three weeks afterwards she quickened. At the expiration of 18 weeks our author was summoned, and found her in labour. In fourteen hours she was delivered of a fine healthy child. The abdomen was so much diminished after the birth of the child that our author entertained no suspicion of any thing remaining in the uterus besides the secundines, which were expelled half an hour afterwards, when the womb appeared to have contracted to the usual size. Half an hour after this a blighted fœtus, of about four months, dark in hue and fetid in smell, presented itself and came away, together with a putrid placenta.

"On reviewing the circumstances of this case, I must observe, that whatever may be inferred from some extraordinary accounts on record, no aid to the doctrine of superfœtation is derivable from this instance; and I think there can be no doubt that the exertion and fright before-mentioned, occasioning the pain and other symptoms, produced also the partial separation of the less vascular perhaps, or more slightly attached placenta, and, through that medium, the death of the smaller and less vigorous fœtus; an event marked by the rigor, coldness within the belly, and sense of weight above spoken of." 327.

The practical inference, our author observes, to be drawn from this case, appears to be that where, at any period of pregnancy, there have been symptoms of abortion, although they have perfectly subsided, the accoucheur should, during parturition, bear in mind the possibility of there being a dead as well as a living child, and consequently institute a more rigorous examination than, at the time alluded to, he had believed to be necessary.

Art. V. *Two Cases of Obstruction of the Colon; in one of which, Organic Affection of the Aorta and Heart was suspected—and, in the other, proved to exist.* By W. C. Callow, Esq. Surgeon, late of the 20th Dragoons.

In September 1821, our author was summoned, at midnight, to a young lady, 19 years of age, who suddenly became insensible, and was thought to be expiring. For three or four days previously she had complained of head-ache and sickness at stomach, to relieve which she had taken an emetic and some aperient. Mr. C. found her in complete coma, with pale face, dilated pupils, laborious and slow respiration, quick feeble pulse, and cold extremities. There being distention of the abdomen, a brisk cathartic was administered—a blister to the scalp—heat in the stomach and extremities. These measures producing some relief, it was deemed proper to bleed next day from the arm, but the loss of a few ounces produced fluttering of the pulse. Eight ounces, however, were abstracted, when the patient indistinctly exclaimed that she was better. By perseverance in similar measures the symptoms were dissipated; but it was three weeks before the young lady could raise her hand to her head, and several more before she regained the muscular power of her lower extremities. She was now seized with what Mr. Callow considered to be "acute hepatitis"—"the liver, upon examination, presenting itself below the margin of the ribs, indurated, and apparently so enlarged, that it could be traced from the right of the hypochondrium to the left of the epigastrium." Calomel and opium, followed by castor oil, brought away "copious evacuations of black, broken-down scybalæ, mixed with portions of fæces of recent formation." The measures which Mr. C. pursued on this occasion are stated to have been successful, the symptoms progressively giving way, and the patient daily indicating the return of health. In a short time, only a very small portion of the hepatic enlargement was visible. Attacks of syncope now came on, with great acceleration of pulse, and constant head-ache. Next came a train of pulmonic symptoms, ending in purulent expectoration, still attended with attacks of syncope and palpitation. Death closed the scene, but permission could not be obtained for opening the body. From many cases which we have seen, and from the case which is now about to be narrated, we think there is not the least doubt that the supposed enlargement of the liver was no other than accumulation in the transverse arch of the colon. Enlargements of the liver, so palpable as that described above, are not to be reduced so suddenly (if at all) as appeared to be the case in Mr. Callow's patient.

*Case* 2. This was the sister of the abovementioned patient, who was suffering from an incessant, hard, dry cough, with constant head-aches ,sickness at stomach, and obstinately constipated bowels. *Strict diet, cathartics, pediluvium prescribed.* The alvine evacuations were found to be very depraved. The abdomen, on examination, felt tumid and unequally distended.

"In a few days the discharge from the bowels became much more copious, but each evacuation was attended with very great pain in the lower part of the abdomen, and a troublesome tenesmus. The dejections consisted mostly of hard, black scybalæ, mixed with fæces evidently of recent formation." 331.

Yet the cough and head-ache remained with little abatement. Venesection to 18 ounces—calomel and antimony exhibited—castor oil daily.

"Under this treatment, some trifling amendment in the violence of the cough was observed, but the bowels were moved with difficulty, never without pain, and generally with tenesmus. A feeling of distention was complained of, as if the contents of the bowels were not carried off, and the abdomen was more tumefied generally. The feet were invariably cold, the head in constant pain, and the right side of the face frequently swollen." 332.

Calomel and opium, succeeded by oil of turpentine and castor oil, brought off a copious discharge of black fetid fæces, and small hard scybalæ, each evacuation being attended with great pain. We cannot follow our author through all his details of symptoms and treatment. We shall give an extract from his report in the month of March, which ought to be read with attention.

"After a perseverance in this kind of treatment for some weeks, the patient one morning complained of an acute pain under the margin of the ribs of the right side, extending anteriorly to the ensiform cartilage, and posteriorly to the spine. Upon examination a considerable enlargement presented, occupying the hypocondrium, and extending towards the middle of the epigastrium. This induration had exactly the feel of the tumid margin of a diseased liver, and was precisely situated where that viscus is to be felt, when morbidly enlarged. The alvine discharges had, from the first, demonstrated great derangement in the hepatic organs, and excessive irregularity in the secretion of bile. The almost daily exhibition of aperients, with the frequent and regular use of cathartics, for the last four months, had, I presumed, rendered it impossible there could be any accumulation in the colon. I concluded, therefore, after some reflection, that the disease now existing, was a highly morbid state of the liver, and that the whole train of symptoms my patient had suffered from, were merely effects." 333.

Under the full impression of the disease being of a hepatic nature, our author commenced that treatment which he considered best adapted for the complaint. This treatment is not specified. It did not, however, succeed. About two months subsequently the pain in the chest became very acute in the region of the heart—and, upon examination, three of the ribs appeared considerably elevated. "The beat of the heart could be distinctly heard and counted. A pulsation could be seen and felt in epigastrio." Spinal disease was discovered soon after this, and relieved by caustic issues. In September the report is, that debility is making rapid strides—that there is decided pressure on the brain, and evident organic disease of the heart. The alvine excretions were now more easily procured, and they were of a more healthy character. "No scybalæ nor hardened fæces had passed for months; still the abdomen was tumid, with a feel of great inflation, and the patient was troubled with constant eructations, and a formation of gastric acid." Death closed the scene in October.

"*Dissection.* Upon dividing the parietes of the abdomen, a portion of the colon forcibly protruded, and upon my continuing the incision to the pubis, not any thing but colon, thinly covered with pale, bloodless omentum, was to be seen. Upon removing the omentum, and proceeding in my examination, I found the colon enormously distended, had occupied nearly the entire cavity of the abdomen, displacing or compressing the other viscera, in an almost incredible manner. The small intestines were pressed down into the cavity of the pelvis, where, like a coil of small rope, they lay in contact with the uterus, void of contents, and perfectly white. The stomach was diminished to the size of a large pear, its muscular coat greatly thickened and unusually hard, with the villous coat highly vascular, and of a light red colour. *The liver had been closely impacted against the diaphragm, with which it had formed two adhesions, was diminished to about half its usual size, but no morbid appearance could be detected in its substance, nor evidence of any having ever existed, with the exception of the adhesions of the serous membrane to the concave side of the diaphragm.* The gall-bladder was large, and distended with bile. The spleen was extremely diminutive, but unusually firm in texture. The pancreas was thin as a riband, and with difficulty distinguished from peritoneum. The kidneys did not present any morbid appearances.

"Tracing the colon from the small intestines, its enlargement was apparent immediately above the cæcum, increased considerably as it ascended, was excessive in its transverse arch, and in its descent, till it reached below the crest of the left ilium, where a kind of cul de sac had been formed, immediately below which it was so much diminished as to be with great difficulty distinguished by the eye from the folds of the peritoneum; but between the finger and thumb the sigmoid flexure could be traced crossing an unusually acute angle formed between the last lumbar vertebra and the sacrum. Here the bowel presented to the touch the resemblance to a small hard inelastic cord, till its entrance into the rectum, which in itself was of less than the usual magnitude, but not considerably so.

"Having divided the colon, in its whole length, from its mesenteric attachments, and passed a ligature at the cæcum, it became evident that this amazing intestine was occupied only by flatus. Upon completing the dissection of the rectum from the vagina and sacrum, I removed the colon from the abdomen for the purpose of a minute inspection of the whole canal.

"The anus was surrounded by a small, deep, corroding ulcer, but it occupied only the integuments of the margin, and did not enter the rectum. The mucous membrane of the rectum was much thickened in places, puckered up in folds, corroded in patches, and covered with a thick tenacious coat of muco-purulent fluid.

"The colon suddenly diminished immediately above the rectum, and at and about the sigmoid flexure, more especially where it had lain in contact with the superior margin of the sacrum, the calibre of the bowels would not more than admit the point of my little finger. For about twelve inches in length the villous coat of the intestine bore the appearance of long-continued increased action, being corroded in several long, narrow furrows, and the canal filled with a tenacious purulent secretion. The muscular coat was much thickened, and in such a state of scirrhous consistence, that in slitting it open the scalpel met with a resistance similar to dividing cartilage. Within the ala of the ilium, and immediately above the stricture, the bowel had obtained the almost incredible size of fourteen inches circumference, perfectly void of fæces, as white in appearance as writing paper, and the coats so extenuated as to be almost as thin. From this part to the left hypochondrium there was some diminution of the calibre of the bowel, which had formed a number of irregular pouches; but the whole of the transverse arch, and most of the ascending portion, was enormously enlarged, of a pallid whiteness, and delicate texture. Scarcely any fæces were contained in the bowel, except in the ascending portion, and those were fluid, and not of unhealthy appearance. Not any thing remarkable was to be observed in the remaining part of the intestinal canal, except that the small intestines were unusually white, and of diminutive appearance. The bladder and uterus were of perfect formation and healthy structure." 338.

On opening the thorax the pericardium was found containing nearly a pint of deep yellow serum. The heart was greatly enlarged, fatty, and flaccid, "presenting the appearance of parboiled meat." The left ventricle was very much enlarged, its parietes thin and weak. The opening into the aorta was of great magnitude, the semilunar valves being quite incapable of closing the orifice. The aorta itself was twice its natural calibre, and this enlargement increased as the aorta was traced downwards. At the crura of the diaphragm the vessel measured six inches in circumference.

"Reverting again to the abdomen, I proceeded to lay bare the artery upon the spine, when I discovered a hollow, or rather flattened part of the vertebræ, occupying about four inches of the column. The artery here, immediately after its exit from the diaphragm, had obtained a sudden contraction formed like the shoulder of a French wine-bottle. The vessel beneath the contraction had shrunk to a very diminutive size, and was perfectly empty, as were all the vessels given off from it. The two external iliacs were quite vacant, and not larger than crow-quills." 339.

On examining the spine it was found that a considerable loss had been sustained in the bodies of three of the vertebræ (lower dorsal and upper lumbar), apparently by absorption, there being no caries. There was no disease in the lungs.

Both these ladies had enjoyed uninterrupted good health till within twelve months of the time our author had them placed under his care. Each of them traced the derangement of her health to constipation of the bowels. The family consisted of four benevolent and affectionate sisters, who had, for many months, indiscreetly cofined themselves to the house in the sedentary occupation of needlework for the poor. Two of them appear to have fallen sacrifices to their unreasonable philanthropy. A third has recently escaped a similar catastrophe from sudden and total obstruction of the colon, requiring the most decisive measures for its removal. The fourth has an enlargement of the thorax on the left side—a distressing feeling in the chest—irregularity of pulse—and incipient stricture of the œsophagus.

There can be no doubt, we think, that a strong predisposition to organic disease must have existed in the constitutions of these four sisters, otherwise the sedentary occupations abovementioned, and to which thousands are exposed for years with little bad effect, could never have produced such dreadful consequences. This last case shews how medical men may be deceived by mistaking accumulations in the colon for organic diseases of the liver. The case also strongly points out the necessity of examining the state of the abdominal viscera in obscure and anomalous diseases. We have had unequivocal proofs in our own practice that scybalæ will lurk in the cells of the colon for many months, although fæces are daily discharged with regularity from the rectum. The irritation which these excrementitious bodies produce on the organic structure with which they lie in contact, gives rise to a host of anomalous affections seated apparently at a great distance from their real causes.

The thanks of his brethren are due to Mr. Callow for this communication.

ART. VI. *An unusual Case of Twin Conception and Labour; also, a Case of blighted Ovum, which was retained in the Uterus eleven months; with Practical Observations, &c.* By JOHN POWELL, Esq. Surgeon and Accoucheur to the Lying-in Institution, Newman Street, London.

*Case.* A lady fell in labour of her ninth child, on the 26th February 1820, and continued with labour pains for the space of 48 hours. Our author then ruptured the membranes, a large quantity of liquor amnii escaping. In two hours the child was delivered. A violent hæmorrhage supervened, but was restrained as soon as the placenta was brought away and regular pressure made. The womb appeared to be contracted to the usual size. On introducing the finger into the vagina to feel if any shreds of membrane remained, our author was surprised to find "a hard irregular substance enveloped in membrane, resembling the bones of a fowl," and which, when brought away, proved to be a perfectly formed fœtus, apparently of about four months, squeezed flat, and without any marks of putridity. Its placenta was found detached within the uterus, but shewed no umbilical vessels ramifying on its surface, being (instead of a loose spongy mass) converted into a firm, fleshy, somewhat tuberculated substance. Our author believes this to be the only case upon record, "where a blighted fœtus has not been expelled in a putrid state, and in which the rationale of the occurrence is satisfactorily accounted for, without having recourse to the doctrine of superfœtation." Superfœtation, in the human subject, he believes, to be almost impossible, except where there is a double uterus. Although cases similar to the above are related by different authors, the appearances of the placenta are not mentioned by any writer, as far as Mr. P. is acquainted, which, he thinks, is surprising, considering its morbid state in the present instance. This morbid state is dwelt upon in cases of blighted ovum of single conceptions, a case of which Mr. Powell digressively relates, as connected with the theory which he means to illustrate. The particulars of the case were as follows:—

A young healthy woman was married in March 1817, having previously menstruated regularly. She became pregnant, about the month of May, and was threatened with abortion at the term of three months, but by care she went on till within eight or ten days of delivery, when a slight flooding accompanied by pain took place, and continued more or less till her accouchement, which occurred on the 31st of March 1818. The labour pains were violent, and it required depletion to prevent puerperal convulsion.

"At length a fleshy mass was expelled from the uterus, about the size of a four months placenta, but as much more dense in structure as in the former case, and bestudded on its internal surface with an appearance of hydatids. The woman did well; but after the expiration of a month, she discharged nearly a pint basin full of small hydatids, which continued coming away for many days; since which she has menstruated regularly, except when pregnant, and has had several children, without any of her former anomalous circumstances. *The growth of the fœtus being here destroyed at an early period, it was probably very small, and escaped unobserved with the coagula, that occasionally were discharged in the week prior to the commencement of labour, as I did not see the whole that came away, not visiting her every day*; however, that the substance which ultimately came from her was a placenta, was quite evident, and cases of this nature not being very uncommon, though generally terminating sooner, the facts will be readily admitted." 347.

Considering that this case is brought forward for the support of a theory, we confess that the proof of the "fleshy mass" being a placenta, appears to us to be exceedingly weak —and as for the proof of the accompanying fœtus, it is altogether wanting. Is it likely that the fœtus itself should have escaped unobserved with the coagula, while the placenta produced such violent parturient efforts for its expulsion?—The question of superfœtation we shall not here enter upon. From the well authenticated *facts* brought forward by Foderé in particular, and the recent case published by Dr. Maton, we are inclined to believe in the possibility of such an event, notwithstanding the ingenious *arguments* opposed to it by various authors.

In conclusion, Mr. Powell offers some practical suggestions to the junior part of the profession, respecting the danger of leaving a patient with a second fœtus in utero—a circumstance which occurred, he observes, in the practice of a gentleman of "first rate talents" a few years ago in this metropolis.

Mr. P. makes it a rule to introduce two or three fingers into the vagina, for the purpose of removing any portion of membrane, &c. which may accidentally have been torn off in withdrawing the placenta, he having known the retention of such substances keep up irritation and pain, with an increased fœtor and discharge of the lochia, for many days.

### Art. VII. *A Case of Cut Throat, successfully treated.* By C. T. Haden.

We dislike the introduction of vulgar and coarse terms in medical or surgical science. They are introduced by some people, under the idea of simplifying the language, and banishing what they call "jargon" from medical writings. Thus we shall have "broken head" instead of fractured cranium—"bloody nose" instead of epistaxis—"black eye" instead of ecchymosis palpebrarum—and "cut throat" instead of wounded larynx or trachea. Certainly pedantry is preferable to this—the real jargon which they complain of.

This case was published because Mr. H. thought it involved some peculiar principles of surgical practice important in such events. For these peculiar principles he candidly acknowledges himself indebted to Mr. Alcock.

*Case.* A woman, after suffering pain in her head for some days, but apparently without any thing like reason for the deed, attempted suicide in July 1822, by "dividing the thyroid cartilage and adjacent parts, so as to lay open the larynx and expose to view the posterior surface of the pharynx." The wound of the cartilage was jagged, and a portion of it nearly detached. There was considerable hæmorrhage, but no vessel required ligature except one near the surface. There was violent cough, and occasional attacks of suffocation, which were relieved by clearing the wound, from time to time, of the ropy mucus which accumulated there.

"The blood which continued to flow, or rather to ooze rapidly from the divided surfaces, for a considerable time, was prevented from being carried into the windpipe during the inspirations (which were, as might be expected, much more violent than natural), by inclining the body forwards, and by the constant and cautious application of soft sponges, frequently renewed, immediately after being squeezed from hot water." 352.

After the cessation of the hæmorrhage, the wound was carefully cleaned, and the fragments of cartilage removed. Two ligatures were then introduced, but not drawn tight—nor were the parts closed, except occasionally, for two hours, on account of the sticky mucus. Drink taken by the mouth came principally away through the wound, producing cough and threatening suffocation. In two hours the wound looked pale and free from blood. The ligatures therefore were drawn so that the lips of the wound were lightly but accurately closed. Compresses and slips of adhesive plaister were applied secundum artem. There was an aperture left in the middle for the expiration of sputa, to which aperture a sponge was applied in the intervals of the cough. All food and drink were strictly prohibited for 30 hours, when a little milk was sucked through a straw, but passed almost entirely through the wound. *Third day.* Nearly one half of the wound was observed to be united; but the other half was wider than it ought. A little milk seemed to pass into the stomach. On the *fourth* day there was reaction that required bleeding. The edges of the wound were brought together by another ligature, and sticking-plaister with compresses were applied. From this period the case went on well; and in a fortnight the wound had become contracted to a small size, though liquids still passed through it in deglutition.

In a month, there was only a small fistulous opening. This ultimately closed entirely.

It is properly observed in a note to the above case, that few wounds penetrating the larynx or trachea admit of the attempt to close the opening, until after the irritation consequent on the wound has subsided. The accumulation of tenacious mucus is so great, in many instances, as not only to endanger, but actually to produce suffocation, unless precautions be taken to moderate it by allaying irritation, and removing the mucus as fast as it is accumulated.

One of the dangers, in cases of this kind, is hæmorrage; and the consequences of bleeding would be enhanced if the wound were tightly bound up. A case is related in a note, which we shall extract as a warning.

"A gentleman cut his throat in the night. The wound was very large; but as the hæmorrhage had ceased, the two attendant surgeons closely stitched up the wound, and left their patient. At an early hour the next morning the poor man was dead. The writer witnessed the dissection; and he considered that death took place in consequence of the immense sanguineous engorgement of the cellular membrane surrounding the wound. Secondary hæmorrhage had of course taken place; and as the careful closing of the wound had prevented the escape of the blood, the latter had been so forcibly injected into the surrounding parts, that the whole mass on being divided put on the appearance of a clot of blood. The force of the injection had indeed been surprisingly great. In this case the wound had not penetrated either larynx or trachea." 358.

We once saw a remarkable case of wounded trachea and œsophagus at Pulo Penang. A Malay won all the money which his companion possessed, by gambling. The loser said nothing at the time, but watching an opportunity in the night, he drew a knife across his friend's throat, severed the wind-pipe between the thyroid and cricoid cartilages, and wounded largely the œsophagus behind. The carotid arteries escaped, but the hæmorrhage was great, and the wound was of frightful extent. Nothing whatever was done but keeping the wound clear with a sponge. The man was nourished by milk and broth glysters for more than a fortnight, during which time he could swallow nothing without danger of suffocation. The wound granulated and slowly closed. We believe that the cure might have been accelerated by stitches after the first period of irritation was over. We would also suggest that, in such cases, fluid nutriment should be introduced into the stomach through an elastic tube. We shall close this subject with the following quotation from a note, which we suspect did not emanate from Mr. Haden himself.

"This principle of allowing wounds to remain open for some time after infliction, before they are closed, is abundantly applicable to operations in general. An irritable state exists in all wounds, as the immediate consequence of the first violence. This irritability subsides in a short time. Now union by the first intention will more certainly take place if a wound be tranquil when closed, than if it be irritable; not to repeat, that as the arteries of the part show, by their increased pulsation, that they partake of the irritability, one chance of secondary hæmorrhage is removed, if the irritability be allowed to subside before the wound is closed; for, even the mere pressure of irritable arteries might greatly increase their already exalted local action, and thus induce them to bleed again." 360.

ART. VIII. *Case of Poisoning by Opium.* By J. HAYES, Member of the Royal College of Surgeons.

Mr. Hayes was called to a lady within half an hour from the time she had swallowed about thirteen drachms of laudanum. He found the patient lying on her back in a state of stupor, with the mouth half open, and eyes shut, countenance ghastly, lips livid. The pupils were found to be greatly dilated, tunica conjunctiva reddened, and her whole aspect like that of a person recently much convulsed, and now dying. Two drachms of sulphate of zinc were dissolved in a small teacup-full of warm water, and a small quantity was, with much difficulty, got down the throat—not perhaps exceeding ℈j. of the sulphate. After some time another quantity was got down, and vomiting was produced, the ejected matters smelling of opium. Deglutition now became more easy, and more of the sulphate was exhibited, which was succeeded by more copious vomiting. There were shortly symptoms of determination of blood to the head, and they were relieved by sanguineous depletion. After the laudanum was considered to be completely evacuated, acids and coffee were administered—and the patient recovered. The treatment was very judicious, according to the then known means; but the injecting apparatus will now supersede all others, especially in cases of poisoning by tincture of opium.

ART. IX. *Remarks on the Neuralgia, as a Class of Diseases with an allusion to a Case of Aphonia in Illustration.* By DAVID UWINS, M.D.

Dr. Uwins, as usual, prefaces the principal matter of the paper with sly cuts at the "digestive organ theory," the "vascular theory," and some other theories of the day; but these we shall pass over without being so cynical as the author himself on his own production. "What will my readers say to me for thus rending mountains, and then displaying nothing but the *ridiculus mus* of a case of aphonia treated successfully by galvanism and nitrate of silver?—*vox* et preterea nihil." 372.

In our apprehension the worthy Doctor has not done so much mischief to the mountains as his too tender conscience accuses him of—and therefore we shall proceed at once to the "vox," so speedily retuned by the galvanic battery of Mr. Le Beaume.

A young lady of delicate frame, strumous habit, and nervous susceptibility, was sent up to our author from the country, for medical assistance. She could not articulate in tones higher than "*a very inaudible* whisper." Query, what tone is that which is *inaudible* ?—In this state she had been for four months, although she had been put upon an alterative plan of treatment, "under the supposition of digestive organ derangement," and subsequently taken chalybeates. In the present instance, our author judged the complaint to be purely nervous, because the subject of it, though decidedly strumous, had no marks of disordered secretions impeding the vocal powers.

"In a word, I considered the altogether of the case to be nearly similar to the effect that would have been produced by the actual division of the vocal nerves, and that, therefore, the indication of treatment was principally that of restoring nervous excitation. And what more effectual means present of so doing, than imitating Wilson Philip in his operation upon maimed animals? Under this feeling I ordered her to be galvanized; the operation was performed by Mr. Le Beaume: he applied one end of the wire to the epigastrium, and the other to the cervical vertebræ, and, in the first instance, only used a very small number of plates from a large trough. In conjunction with the galvanism I prescribed pills composed of a sixth part of a grain of nitrate of silver, which I gradually increased to half a grain. In two days from the commencement of the plan, the voice was decidedly improved; it was not, however, completely restored till after the lapse of about a week; and this gradual restoration I, of course, hailed as a more satisfactory result than had the recovery been immediate." 374.

We think that those practitioners who are in the habit of exhibiting the nitrate of silver, will not attribute much of the cure to doses of a sixth of a grain up to half a grain for a week. The fact is, that there is something very unaccountable in these cases of aphonia. They come on without apparent cause—they continue, in many instances, to resist every remedy—and, lastly, go off, where no medicine is taking, as suddenly as they came on. Having often seen these instances, we attach little importance to the disappearance of the complaint, in a single case, while under a galvanic treatment. At the same time, as the accumulation of facts must begin with some single one, we lay Dr. Uwin's case before our readers with the view that—"*valeat quantum valere debet.*"

The last paper in the volume is one by Mr. Alcock, on congenital division of the palate, which we cannot analyze without reference to a well-executed plate, and therefore refer our readers to the original.

We have now closed the first volume of the Surgeon-Apothecaries' Transactions. We hope the first will not be the last. We advise the council of publication to take care not again to admit so much heterogeneous matter as this volume contains. No work could prosper, or at all exist under such circumstances. The papers, in a publication of this kind, should not be so long and prosing as many of these are. Well attested facts and concise observations on them, are the materials which are wanted, and the only ones that will afford healthy nutriment for the periodical volumes of a society or association.

## Figures and Tables

**Figure f1:**